# Combined Biomarker Analysis for Risk of Acute Kidney Injury in Patients with ST-Segment Elevation Myocardial Infarction

**DOI:** 10.1371/journal.pone.0125282

**Published:** 2015-04-08

**Authors:** Ying-Chang Tung, Chih-Hsiang Chang, Yung-Chang Chen, Pao-Hsien Chu

**Affiliations:** 1 Department of Cardiology, Chang Gung Memorial Hospital, Taipei, Taiwan; 2 Department of Nephrology, Chang Gung Memorial Hospital, Taipei, Taiwan; 3 Healthcare Center, Chang Gung Memorial Hospital, Taipei, Taiwan; 4 Chang Gung University College of Medicine, Taipei, Taiwan; Biogen Idec, UNITED STATES

## Abstract

**Background:**

Acute kidney injury (AKI) complicating ST-segment elevation myocardial infarction (STEMI) increases subsequent morbidity and mortality. We combined the biomarkers of heart failure (HF; B-type natriuretic peptide [BNP] and soluble ST2 [sST2]) and renal injury (NGAL [neutrophil gelatinase-associated lipocalin] and cystatin C) in predicting the development of AKI in patients with STEMI undergoing primary percutaneous coronary intervention (PCI).

**Methods and Results:**

From March 2010 to September 2013, 189 STEMI patients were sequentially enrolled and serum samples were collected at presentation for BNP, sST2, NGAL and cystatin C analysis. 37 patients (19.6%) developed AKI of varying severity within 48 hours of presentation. Univariate analysis showed age, Killip class ≥2, hypertension, white blood cell counts, hemoglobin, estimated glomerular filtration rate, blood urea nitrogen, creatinine, and all the four biomarkers were predictive of AKI. Serum levels of the biomarkers were correlated with risk of AKI and the Acute Kidney Injury Network (AKIN) stage and all significantly discriminated AKI (area under the receiver operating characteristic [ROC] curve: BNP: 0.86, sST2: 0.74, NGAL: 0.75, cystatin C: 0.73; all *P* < 0.05). Elevation of ≥2 of the biomarkers higher than the cutoff values derived from the ROC analysis improved AKI risk stratification, regardless of the creatine level (creatinine < 1.24 mg/dL: odds ratio [OR] 11.25, 95% confidence interval [CI] 1.63-77.92, *P* = 0.014; creatinine ≥ 1.24: OR 15.0, 95% CI 1.23-183.6, *P* = 0.034).

**Conclusions:**

In this study of STEMI patients undergoing primary PCI, the biomarkers of heart failure (BNP and sST2) and renal injury (NGAL and cystatin C) at presentation were predictive of AKI. High serum levels of the biomarkers were associated with an elevated risk and more advanced stage of AKI. Regardless of the creatinine level, elevation of ≥2 of the biomarkers higher than the cutoff values indicated a further rise in AKI risk. Combined biomarker approach may assist in risk stratification of AKI in patients with STEMI.

## Introduction

Acute kidney injury (AKI) is well recognized as an important complication in patients with acute ST-segment elevation myocardial infarction (STEMI). Previous studies reported a high incidence of AKI in acute myocardial infarction (AMI), ranging from 10 to 27% [1–6], and the incidence could be as high as 50% if AMI is complicated by cardiogenic shock [7]. Development of AKI after AMI predicts short- [8] and long-term mortality [1–5, 9] and other major cardiovascular outcomes [10]. In an observational study of the Medicare patients, severe AKI (>1.0 mg/dL increase in serum creatinine) occurring after AMI was comparable in strength with other correlates of cardiovascular mortality, such as diabetes, cerebrovascular and peripheral vascular diseases [1]. Furthermore, even 0.1 mg/dl increase in serum creatinine level after AMI increases long-term risk of end stage renal disease (ESRD) [11]. Therefore, early detection of AKI in STEMI patients may assist in risk stratification and tailoring treatment during hospitalization and follow-up care.

Pathophysiologic interconnections between the heart and kidneys, the cardiorenal syndromes (CRS), are defined as ‘disorders of the heart and kidneys whereby acute or chronic dysfunction in one organ may induce acute or chronic dysfunction of the other [12]’. Type 1 CRS, characterized by acute worsening of cardiac function leading to AKI, often complicates acute coronary syndrome (ACS) and acute decompensated heart failure (ADHF) [13–16]. Acute deterioration of cardiac function may set off a series of changes in neurohormonal and hemodynamic factors leading to arterial underfilling and venous congestion. Inadequate renal perfusion, increased intra-abdominal pressure and passive kidney congestion may culminate in AKI [15–17]. In patients with cardiac diseases or undergoing cardiovascular surgery, new markers of renal dysfunction, such as neutrophil gelatinase-associated lipocalin (NGAL) [18–21] and cystatin C [22–26], have emerged as early markers of AKI prior to any elevations of serum creatinine and have been shown to provide additional prognostic capacity in these settings. Levels of traditional cardiac markers, such as natriuretic peptides [14, 27–29] and troponins [27, 30–32], rise in both cardiac and renal dysfunction, suggesting a bidirectional and reciprocal nature of the vicious circle of CRS.

ST2 (suppression of tumorigenicity 2), consisting of a trans-membrane ligand (ST2L) and a soluble form (sST2), is a member of the interleukin-1 receptor family and has been identified as a novel HF markers in response to mechanical stress [33–37]. As a decoy receptor of interleuin-33 (IL-33) that attenuates its cardioprotective properties [34], serum levels sST2 rise in various cardiac diseases and have been shown to independently predict mortality and other adverse outcomes in HF [38–43] and MI [44–47]. Despite the well-established prognostic role, studies on the role of sST2 in CRS are sparse [48–50]. Compared with HF, few studies on CRS have addressed the development of renal dysfunction in STEMI. This study aimed to combine the biomarkers of HF (BNP and sST2) and renal injury (NGAL and cystatin C) at presentation in the prediction of AKI in STEMI patients undergoing primary percutaneous coronary intervention (PCI).

## Methods

### Study population

From March 2010 to September 2013, patients sequentially admitted to Linkou Chang Gung Memorial Hospital in Taiwan with a diagnosis of STEMI were enrolled in this study. STEMI was diagnosed according to the established criteria [51]. The inclusion criteria were patients with STEMI who presented within 12 hours of symptom onset and received primary coronary intervention (PCI). Exclusion criteria were as follows: STEMI patients presenting longer than 12 hours after symptom onset or not receiving primary PCI, history of renal failure requiring dialysis or with previous kidney transplantation, and unable to provide informed consents. The study protocol complied with the Declaration of Helsinki and was approved by the Institutional Regulation Board of Chang Gung Memorial Hospital, Taiwan (No. 101-5312B). Written informed consent was obtained from all patients.

### Data collection and biomarker assay

All the STEMI patients were enrolled at presentation to the emergency department. The collected data included age, gender, body mass index (BMI), blood pressure, heart rate, smoking status, and underlying diseases such as coronary artery disease (CAD), diabetes, hypertension, heat failure and hyperlipidemia. The Killip criteria were used during physical examination to classify the severity of STEMI. Patients in Class I demonstrated no evidence of HF. Patients in Class II had findings consistent with mild to moderate HF. Patients in Class III demonstrated overt pulmonary edema and patients in Class IV were in cardiogenic shock [52]. The amount of contrast medium and the stent used in the PCI procedure were recorded. Coronary arteries with lesions of ≥50% diameter stenosis were regarded as diseased, and the number of diseased vessels was recorded. All the patients underwent echocardiography on admission and the left ventricular ejection fraction (LVEF) was acquired by modified Simpson's method.

Blood samples were collected at presentation for basic laboratory tests, including troponin I, complete blood count, blood urea nitrogen (BUN), creatinine, sodium, potassium, and alanine transaminase. The blood samples were then centrifuged at 1,500 rpm for 5 minutes and stored at -80°C until thawed for assay for the biomarkers of HF (BNP and sST2) and renal injury (NGAL and cystatin C). BNP was assayed with the fluorescence immunoassay device Triage BNP Test (Biosite Diagnostics, San Diego, California). Serum sST2 concentrations were measured with the use of a high-sensitivity sandwich monoclonal immunoassay (Presage ST2 assay; Critical Diagnostics, San Diego, California). NGAL and cystatin C were measured by a single enzyme-linked immunosorbent assay (R&D Systems, Minneapolis, Minnesota).

### Renal function assessment

The primary endpoint of this study was the development of AKI within 48 hours of presentation, according to the creatinine criteria of the Acute Kidney Injury Network (AKIN) [53]. Creatinine data were collected at presentation to the emergency department and within 48 hours of presentation. Admission estimated glomerular filtration rate (eGFR) was calculated using Modification of Diet in Renal Disease equation for Chinese patients [54]. The AKIN stage 1 is defined as an increase in serum creatinine of more than or equal to 0.3 mg/dL or increase to more than or equal to 1.5- to 2-fold from baseline. Stage 2 is increase in serum creatinine to more than 2- to 3-fold from baseline. Stage 3 is increase in serum creatinine to more than 3-fold from baseline (or serum creatinine of more than or equal to 4.0 mg/dL with an acute increase of at least 0.5 mg/dL).

### Statistical analysis

Categorical variables were expressed as numbers and percentages and the chi-square test were used to compare variables between the study groups. Continuous variables were expressed as means ± standard deviation and compared with the Student’s t-test. Values of BNP, sST2, NGAL and cystatin C were reported as median (quartile 1—quartile 4) and compared with the Mann-Whitney U test between the study groups. The Kruskal-Wallis test was used to compare continuous variables across multiple groups. The Spearman’s analysis was used to evaluate the correlation between the biomarkers. To determine the predicting factors of AKI, variables that were significant in the univariate logistic regression analysis were then incorporated into the multivariate logistic regression analysis. The receiver operation characteristic (ROC) analysis was used to evaluate the discrimination capacity of the biomarkers. A *P* value less than 0.05 was considered to be statistical significant. Data were analyzed with the SPSS version 19.0 (SPSS, Chicago, Illinois).

## Results

A total of 189 patients presenting with STEMI from March 2010 to September 2013 were included in this study. Among the study patients, 37 (19.6%) developed AKI within 48 hours of admission. Table 1 shows the demographic and clinical characteristics of the AKI and non-AKI groups. There was no significant difference between the two groups in gender, BMI, mean arterial pressure, heart rate, LVEF, eGFR, prior CAD, HF, diabetes, hyperlipidemia, smoking status, number of diseased vessel on coronary angiography, stent deployment, contrast volume, use of medications, and serum levels of troponin I, sodium, potassium and alanine aminotransferase. Compared with the patients without AKI, patients in the AKI group were older (68.14 ± 12.64 years vs. 61.33 ± 13.86 years; *P* = 0.044) and more frequently presented with Killip class ≥2 (81.8% vs. 33.3%; *P* = 0.017) and hypertension (81.8% vs. 49.3%; p = 0.044). The AKI group also had higher mean levels of white blood cell (WBC) counts (12800 vs. 10449; *P* = 0.041), BUN (33.53 vs. 17.89; *P*<0.001) and creatinine (2.00 vs. 1.03; *P*<0.001) and median levels of BNP (1225 vs. 139.5; *P*<0.001), sST2 (72.91 vs. 33.15; *P*<0.001), NGAL (238.5 vs. 86.95; *P*<0.001), and cystatin C (2.4 vs. 0.86; *P* = 0.007), and lower levels of hemoglobin (12.69 vs. 14.18; *P*<0.001) and eGFR (50.13 vs. 90.68; *P* <0.001).

**Table 1:** Baseline characteristics.

	AKI (n = 36)	Non-AKI (n = 153)	*P* value
Age (years)	68.14±12.64	61.33±13.86	0.01
Male gender (%)	90.9	85.5	0.629
Body mass index (kg/m2)	25.7±3.79	25.21±3.91	0.714
Mean arterial pressure (mm Hg)	116.87±140.67	97.84±22.9	0.424
Heart rate (per minute)	83.31±26.6	74.32±17.09	0.163
LVEF (%)	52.3±21.31	57.04±11.85	0.178
Killip class ≥ 2 (%)	81.8	33.3	0.017
**Comorbidities (%)**			
CAD history	36.4	15.9	0.107
Heart failure	9.1	1.4	0.132
Diabetes mellitus	54.5	27.5	0.073
Hypertension	81.8	49.3	0.044
Hyperlipidemia	52.4	70.4%	0.098
Smoking	54.5	60.9	0.691
**Percutaneous coronary intervention**			
Number of diseased vessel (%)			0.884
1 vessel	45.5	42.6	
2 vessels	36.4	32.4	
3 vessels	18.2	25.0	
Stent deployment (%)	83.6	89.7	0.84
Contrast volume (mL)	254.0±32.86	253.67±63.76	0.991
**Laboratory tests, mean ± standard deviation**			
WBC (/mm^3^)	12800±6433	10449±3137	0.041
Hemoglobin (mg/dL)	12.69±2.09	14.18±2.06	<0.001
BUN (mg/dL)	33.53±17.82	17.89±13.79	<0.001
Creatinine (mg/dL)	2.00±1.35	1.03±0.74	<0.001
eGFR (min/mL)	50.13±28.69	90.68±28.48	<0.001
Sodium (mg/dL)	140.0±3.03315	139.23±2.8	0.406
Potassium (mg/dL)	4.04±0.65	3.87±0.38	0.423
ALT (units/L)	54.67±44.94	45.4±30.6	0.511
Troponin I (ng/mL)	7.65±25.33	4.88±6.46	0.169
**Biomarkers of heart failure and renal injury, median (quartile 1—quartile 4)**			
BNP (pg/mL)	1225 (567.5–2120)	139.5(40.64–363.75)	<0.001
sST2 (ng/mL)	72.91 (39.05–130.36)	33.15 (25.55–62.34)	<0.001
NGAL (n/mL)	238.5 (156.06–336.79)	86.95 (62.64–15.45)	<0.001
Cystatin C (mg/L)	2.4 (2.07–2.87)	0.86 (0.63–1.22)	0.007
**Medication (%)**			
Diuretics	33.3	17.5	0.087
DAPT	90.9	95.7	0.503
ACEI/ARB	81.8	79.7	0.871
Beta blocker	81.8	92.8	0.233
Calcium channel block	19	10.5%	0.253
Statin	81.8	91.3	0.33

ACEI: angiotensiogen converting enzyme inhibitors; AKI: acute kidney injury; ALT: alanine transaminase; ARB: angiotensin receptor inhibitors; BNP: B-type natriuretic peptide; BUN, blood urea nitrogen; CAD: coronary artery disease; DAPT: dual antiplatelet therapy; eGFR: estimated glomerular filtration rate; LVEF: left ventricular ejection fraction; NGAL: neutrophil gelatinase-associated lipocalin; WBC: white blood cell.

Table 2 shows the results of the univariate logistic regression analysis. Except for hypertension, all variables with a *P* value <0.05 in Table 1 remained significantly associated with AKI. However, in the multivariate analysis, no variable remained significant in predicting the development of AKI. As shown in Table 3, creatinine significantly correlated with the biomarkers of heart failure and renal injury. There was also significant correlation between BNP and sST2 (r = 0.325; *P* = 0.014) and between NGAL and cystatin C (r = 0.545, *P*<0.001), but the correlation between the biomarkers of HF and renal injury was inconsistent. In Table 4, the results of the ROC analysis revealed that all the four biomarkers significantly discriminated the development of AKI (area under the ROC curves: BNP 0.86, sST2 0.74, NGAL 0.75, cystatin C 0.73; all *P*<0.05).

**Table 2:** Univariate analysis of predicators of acute kidney injury within 48 hours.

	Odds ratio	95% CI	*P* value
Age	1.039	1.009–1.07	0.011
Killip class ≥ 2	4.958	1.297–18.952	0.019
eGFR	0.952	0.931–0.974	<0.001
Hypertension	4.623	0.932–23.018	0.061
WBC	1.001	1.001–1.002	0.009
Hemoglobin	0.712	0.587–0.863	0.001
BUN	1.069	1.025–1.115	0.002
Creatinine	3.701	1.794–7.632	<0.001
BNP	1.002	1.001–1.004	<0.001
sST2	1.004	1.001–1.007	0.006
NGAL	1.004	1.001–1.007	0.003
Cystatin C	1.002	1.001–1.003	0.001

BNP: B-type natriuretic peptide; BUN, blood urea nitrogen; eGFR: estimated glomerular filtration rate; NGAL: neutrophil gelatinase-associated lipocalin; WBC: white blood cell.

**Table 3:** Spearman’s correlation analysis of creatinine and the biomarkers of heart failure and renal injury.

	Creatinine	BNP	sST2	NGAL	Cystatin C
Creatinine	1.0	0.394 (*P* = 0.002)	0.357 (*P*<0.001)	0.376 (*P*<0.001)	0.261 (*P* = 0.002)
BNP	0.394 (*P* = 0.002)	1.0	0.325 (*P* = 0.014)	0.155 (*P* = 0.282)	0.302 (*P* = 0.14)
sST2	0.357 (*P*<0.001)	0.325 (*P* = 0.014)	1.0	0.243 (*P* = 0.055)	0.204 (*P* = 0.011)
NGAL	0.376 (*P*<0.001)	0.155 (*P* = 0.282)	0.184 (*P* = 0.079)	1.0	0.545 (*P*<0.001)
Cystatin C	0.261 (*P* = 0.002)	0.302 (*P* = 0.14)	0.204 (*P* = 0.011)	0.545 (*P*<0.001)	1.0

BNP: B-type natriuretic peptide; NGAL: neutrophil gelatinase-associated lipocalin.

**Table 4:** Receiver operating characteristic curve analysis of creatinine and the biomarkers of heart failure and renal dysfunction in predicting acute kidney injury.

	Cutoff value	AUC	95% CI	*P* value	Sensitivity	Specificity	Youden index
Creatinine	1.24 mg/dL	0.89	0.83–0.94	<0.001	0.76	0.91	0.67
BNP	676 pg/mL	0.86	0.76–0.93	<0.001	0.75	0.89	0.64
sST2	50.77 ng/mL	0.74	0.64–0.79	<0.001	0.71	0.72	0.43
NGAL	133.7 ng/mL	0.75	0.64–0.85	0.006	0.7	0.77	0.47
Cystatin C	1.6 mg/L	0.73	0.63–0.81	0.016	0.79	0.69	0.48

AUC: area under the receiver operating characteristic curve; BNP: B-type natriuretic peptide; NGAL: neutrophil gelatinase-associated lipocalin.

**Fig 1:**
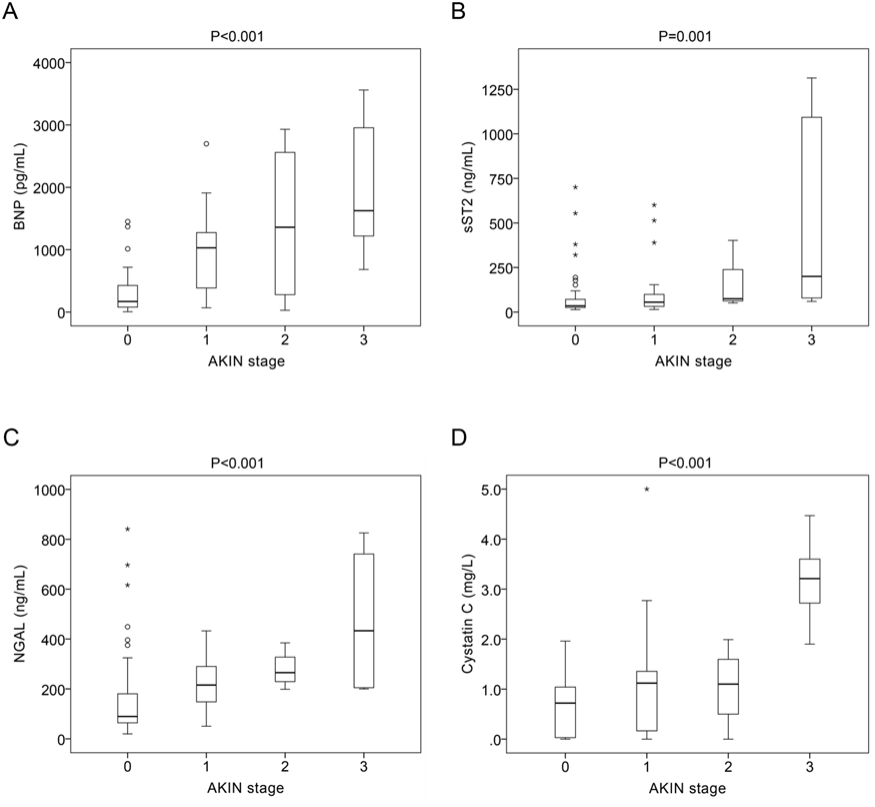
Serum levels of the biomarkers at presentation and the Acute Kidney Injury Network (AKIN) stages in patients developing acute kidney injury within 48 hours. Serum levels of BNP (A), sST2 (B), NGAL (C) and cystatin C (D) at presentation increased significantly across the spectrum of the AKIN stages within 48 hours.

**Fig 2:**
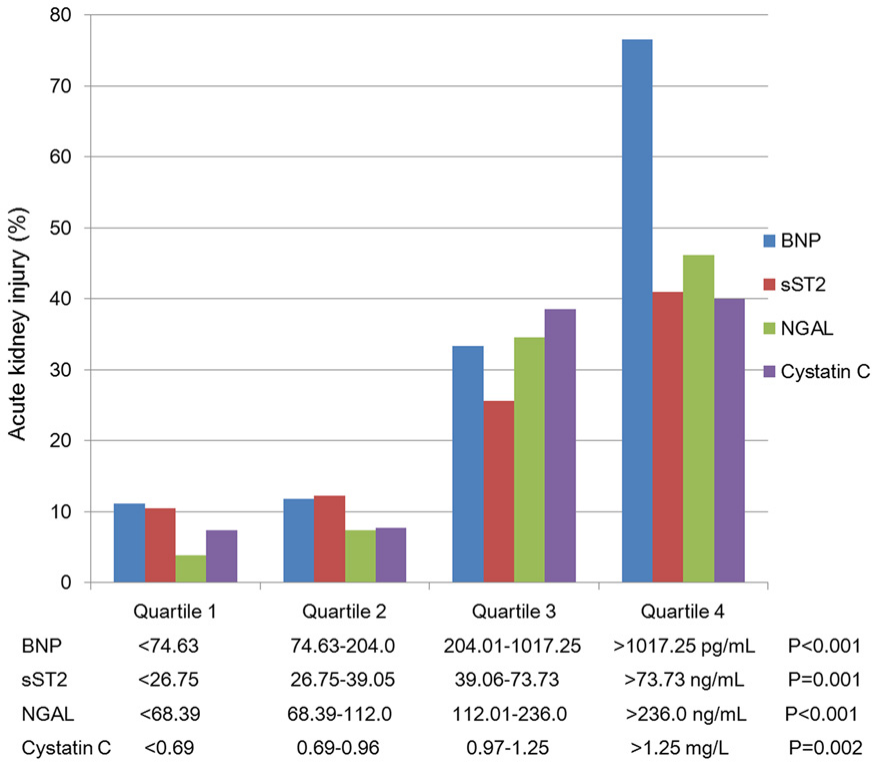
Serum levels of the biomarkers at presentation and the risk of developing acute kidney injury within 48 hours. The risk of developing acute kidney injury at 48 hours increased significantly as serum levels of biomarkers rose at presentation.

**Fig 3:**
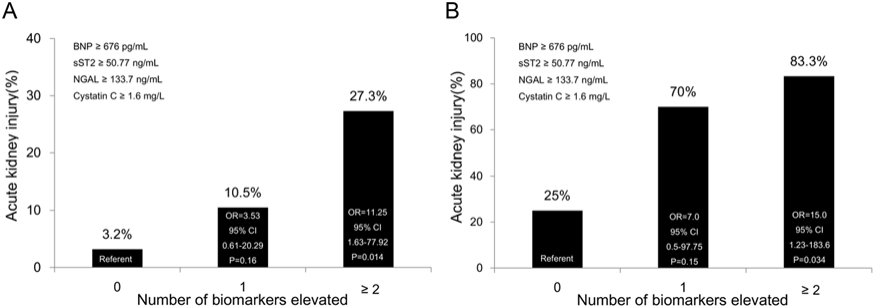
Number of biomarkers elevated higher than the cutoff values and the risk of developing acute kidney injury within 48 hours: (A) patients with creatinine levels <1.24 mg/dL; (B) patients with creatinine level ≥1.24 mg/dL. Regardless of the creatinine level on admission, elevation of ≥2 of the biomarkers higher than the cutoff values provided incremental value in risk stratification of acute kidney injury.

Fig 1 illustrates the association of serum levels of the biomarkers and the AKIN stage. Serum levels of BNP, sST2, NGAL and cystatin C increased significantly across the spectrum of the AKIN stages. The risk of AKI also positively correlated with the serum levels of the biomarkers (Fig 2). Using the creatine cutoff value of 1.24 mg/dL to group the STEMI patients into low and high risk of AKI, a further rise in risk was noted in the both groups if the patients presented with ≥2 biomarkers elevated higher than the cutoff values derived from the ROC analysis (Fig 3). This result implied that elevation of the biomarkers at presentation help discriminate the development of AKI within 48 hours, even in patients considered to be of low risk based on the creatine levels.

## Discussion

In the current study, we aimed to combine the biomarkers of heart failure (BNP and sST2) and renal injury (NGAL and cystatin C) in diagnosis of AKI in patients with STEMI. The main findings of this study could be summarized as:

1. Serum levels of the four markers at presentation were predictive of AKI within 48 hours in STEMI patients undergoing primary PCI.
2. Serum levels of the biomarkers were correlated with an elevated risk and more advanced stage of AKI.
3. Regardless of the creatinine level on admission, elevation of ≥2 of the biomarkers higher than the cutoff values significantly increased the risk of AKI.

Development of AKI after STEMI has been regarded as an important issue in patients with STEMI because of the increased in-hospital and long-term mortality and subsequent risk of ESRD [1–5, 9]. Patients hospitalized for STEMI are subjective to several complications and interventional procedures associated with AKI, such as cardiogenic shock, heart failure, cardiac catheterization, and coronary artery bypass surgery [7, 55–57]. Prior observational studies have explored the associated risk factors of AKI in these settings, including use of contrast agents, diabetes, previous kidney disease, hemodynamic instability, low cardiac output, and volume depletion [58–60]. Deterioration of renal function following acute alterations in cardiac function due to ADHF or ACS is classified as type 1 CRS [13, 15, 16]. Compared with HF, there are few reports in the literature on type 1 CRS in the setting of AMI. It has been proposed that cardiac dysfunction and hemodynamic perturbations could result in systemic underfilling and renal hypoperfusion, leading to further renal injury [16]. Neurohormonal activation, hypothalamic-pituitary stress reaction, and inflammation have also been shown to contribute to type 1 CRS [16]. However, there is increasing evidence that it may be venous congestion rather than arterial underfilling that is associated decreasing renal blood flow and worsening renal function [61–64]. Diastolic LV dysfunction, the earliest change seen after coronary occlusion [65] results in elevated LV filling pressure and central venous pressure. As demonstrated in an early animal model by Winton et al., temporary elevation of central venous pressure can be transmitted back to the renal veins and result in direct renal dysfunction [66]. A more recent study by Damman et al. showed that increased central venous pressure is associated with impaired renal function and mortality in patients with cardiovascular diseases [64].

BNP was predictive of AKI in the current analysis, consistent with the result of recent studies. Akgul et al. reported that BNP was associated with AKI and 6-month all-cause mortality in STEMI patients [67]. In a biomarker analysis of the HORIZONS-AMI trial, Guerchicoff et al. found that BNP levels were consistently higher in the AKI group at baseline, hospital discharge, 30-day follow-up, and 1-year follow-up [68]. BNP, a cardiac hormone released by ventricular cardiomyocytes, elevates in both systolic and diastolic HF in response to ventricular dysfunction and increased myocardial stress [69, 70]. Given its association with capillary wedge pressure and venous congestion, BNP has been demonstrated to be an indirect marker for worsening renal function in ADHF [71, 72]. In patients with AMI, increased filling pressure and ventricular wall stress secondary to myocardial ischemia may be one of the factors behind the elevation of serum BNP levels [73, 74]. We hypothesized that increased BNP levels indicated elevation of central venous pressure and passive renal congestion and could also serve as an indirect marker of AKI in AMI.

The current analysis may be one of the earliest studies to examine the role of sST2 in AKI in patients with STEMI. sST2 is a novel cardiac biomarker complementary to the natriuretic peptides in the risk prediction of HF hospitalization and death [38, 42, 75]. The association between sST2 and AKI discovered in our STEMI cohort may provide another insight into the pathogenesis of type 1 CRS. As a marker of mechanical stretch similar to the natriuretic peptides, sST2 has been demonstrated to significantly correlated with hemodynamics, LVEF, disease severity and adverse remodeling in AMI [44, 46, 47, 76] and predict pulmonary artery pressure, right ventricular hypokinesis and jugular venous distension in ADHF [41]. In a cohort of 3,450 participants from the Framingham Heart Study, serum sST2 concentration correlated significantly with age, diabetes and inflammation [77], which are also regarded as classic risk factors of AKI during acute illness [78]. The association between sST2 and diabetes, hypertension and inflammatory markers not only suggests the role of ST2 as a cardiometabolic marker but implicates its potential in predicting AKI and CRS in acute cardiac events. Importantly, sST2 has been considered to reflect immune cell activation and cell signaling in progressive HF [17]. As a decoy receptor of IL-33, sST2 attenuates cardioprotective effects provided by IL-33 [34], potentially further causing cardiac dysfunction, hemodynamic instability and potentiating the risk of renal injury. Furthermore, serum levels of sST2 have been shown to significantly correlate with neurohormonal activation, as indicated by its association with norepinephrine and aldosterone levels in patient with AMI [76]. On the contrary, however, a recent study in ADHF by Legrand et al. [49] reported lack of association between sST2 and worsening of renal function in patients with ADHF. The difference in study population may be the reason for the inconsistent results. To achieve a better understanding of the association between sST2 and CRS, further study is warranted to delineate IL-33/ST2 pathway in kidney injury in various cardiac events.

Our finding that the biomarkers of renal injury, NGAL and cystatin C, were predictive of AKI is consistent with the results of previous work. Established as biomarkers of AKI, both NGAL and cystatin C have been well explored in cardiovascular diseases beyond confines of nephrology. In a study by Lindberg et al., plasma NGAL independently predicted all-cause mortality and MACE in STEMI patients treated with primary PCI [79]. In that study, higher levels of plasma NGAL was significantly associated with lower eGFR. In patients with acute and chronic HF, serum NGAL has been supposed to be a novel, sensitive marker of worsening renal function [80, 81]. Similarly, cystatin C has also been demonstrated as an early marker of AKI in patients with ADHF [82] and AMI [6, 26] and those admitted to the coronary care unit [83]. Furthermore, an additive prognostic value of cystatin C was found in patients with AMI [26] and ADHF [82].

Given the increased morbidity and mortality associated with the development of AKI in patients with acute illness, there are high demands to establish an ideal biomarker in AKI, such as organ specific, highly sensitive for indicating renal injury, unaffected by other biomarkers, helpful in differential diagnosis and monitoring disease course and response to treatment [84]. Because such an ideal biomarker is probably not available to date, a multi-marker model has been proposed for AKI for risk prediction, early diagnosis and even to give deep insight into pathophysiologic mechanisms. Just as the multi-marker panels used in HF and MI, we hypothesized that combined biomarkers of HF and renal injury may provide incremental diagnostic values and improve risk stratification in STEMI patients developing AKI. Although each of the four biomarkers was predictive of AKI in the current study, however, confounding factors such as age, gender, body habitus and comorbidities may affect the levels of these markers independent of the cardiorenal axis. As a consequence, the specificity of these biomarkers may be somewhat limited. Therefore, we analyzed two classes of biomarkers that potentially play a role in CRS and found that this combined approach may provide additive values in AKI risk stratification, even in the patients with low levels of serum creatinine at admission. Interestingly, while renal function correlated significantly with the biomarkers of HF and renal injury, the correlation between biomarkers of HF and renal injury was inconsistent. A possible explanation of this observation is that impaired renal function in STEMI patients may arise more from hemodynamic alterations and venous congestion than from direct insult to kidney structure. Further investigation of the observed heart-kidney interface in CRS may provide better understanding of the bidirectional nature of pathophysiologic crosstalk in this vicious cycle.

### Limitations

This current study has several limitations. This was a single center study with a small sample number. Along with the few adverse events, we could not provide outcome data such as dialysis or survival. In addition to the biomarkers, there were several factors significantly associated with AKI in this study, such as age, Killip class, renal function, WBC count, and hemoglobin level. Despite the significance in predicting AKI in the univariate analysis, none of these parameters remained statistically significant in the adjusted model. We used serum for NGAL and cystatin C assay but not urine samples, while some suggested that urine NGAL and cystatin C would be more specific to renal tubular injury and dysfunction [49]. It remains to be determined whether analysis with urine biomarkers provides comparable results. Furthermore, the serum levels of biomarkers were measured only at presentation for risk stratification of AKI but not analyzed serially for early diagnosis. All the patients in this study presented with STEMI, and it is unclear whether the results could be generalized to other cardiovascular diseases. Large, prospective multicenter studies are warranted to validate our data and compare it with other exiting biomarkers and parameters in a multi-maker model.

## Conclusions

In this single center study, we found that the biomarkers of heart failure (BNP and sST2) and renal injury (NGAL and cystatin C) at presentation were predictive of AKI within 48 hours in patients with STEMI undergoing primary PCI. High serum levels of the biomarkers were associated with an elevated risk and more advanced stage of AKI. Elevation of ≥2 of the biomarkers higher than the cutoff values indicated a further rise in the risk of AKI, even in the patients with low creatinine levels on admission. Therefore, combination of two classes of the biomarkers that potentially play a role in CRS may provide additive values in AKI risk stratification in patents with STEMI.
